# Transcriptome-Wide Analysis for Ginsenoside Rb3 Synthesis-Related Genes and Study on the Expression of Methyl Jasmonate Treatment in *Panax ginseng*

**DOI:** 10.3390/life11050387

**Published:** 2021-04-25

**Authors:** Kangyu Wang, Zixuan Zhang, Shaokun Li, Jian Hu, Tao Liu, Yang Jiang, Jun Wu, Minghai Lu, Mingzhu Zhao, Li Li, Lei Zhu, Yanfang Wang, Yi Wang, Meiping Zhang

**Affiliations:** 1 College of Life Science, Jilin Agricultural University, Changchun 130118, China; kangyu.wang@jlau.edu.cn (K.W.); zixuanzhang123@163.com (Z.Z.); shaokun.li@case.edu (S.L.); hujian2021420@163.com (J.H.); liutao20210420@163.com (T.L.); wky0427@163.com (Y.J.); w1398608555@163.com (J.W.); luminghai@gensci-china.com (M.L.); mingzhuzhao@jlau.edu.cn (M.Z.); lili@jlau.edu.cn (L.L.); zhulei0916@163.com (L.Z.); 2 Jilin Engineering Research Center Ginseng Genetic Resources Development and Utilization, Changchun 130118, China; 3 College of Chinese Medicinal Materials, Jilin Agricultural University, Changchun 130118, China; yfwang2014@163.com

**Keywords:** *Panax ginseng* C. A. Meyer, ginsenoside Rb3, bioinformatics, functional genes, methyl jasmonate

## Abstract

*Panax ginseng* C. A. Meyer is a kind of renascent herb that belongs to the genus *Panax* in the family *Araliaceae*. It is a traditional Chinese precious herbal medicine with a long history of medicinal use. Ginsenoside Rb3 is one of the important active ingredients in ginseng and has important physiological activity in the treatment of many diseases. In this study, we screened and systematically analyzed the candidate genes related to ginsenoside Rb3 synthesis through bioinformatics methods; discussed the functions, expression patterns, and interactions of the genes related to ginsenoside Rb3 synthesis; and finally, selected seven genes, mainly *PgRb3,* that directly contribute to the synthesis of ginsenoside Rb3. This study provides a reference for revealing the expression rules of ginsenoside Rb3 synthesis-related genes and elucidating the regulatory mechanism of methyl jasmonate, lays a theoretical foundation for the research of ginsenoside Rb3 synthesis, and provides theoretical and technical support for the factory production of ginsenoside monomer saponins.

## 1. Introduction

*Panax ginseng* C. A. Meyer is a perennial herbaceous plant of the genus *Panax* of the *Araliaceae* family. It is also known as stick mallet, earth essence, and divine herb. It is a traditional Chinese precious medicinal material with a long history of medicinal use [1]. Modern studies have shown that the medicinal effects of ginseng mainly include strengthening immunity, regulating the central nervous system, improving heart function, antitumor, antioxidation, and lowering blood glucose. The main components of ginseng include ginsenosides (including more than 50 kinds of ginseng monomer saponins), ginseng polysaccharides, peptides, ginseng alcohol, volatile oil, flavonoids, and many other trace elements [2]. As an economic crop, ginseng has been widely developed and utilized in the fields of food, pharmaceuticals, health care, and beauty [3,4,5].

The main active ingredient in ginseng is ginsenoside, which is a tetracyclic triterpene damarane compound composed of triterpene aglycone, carbohydrate, uronic acid, and other organic acids [6,7]. Dammarane-type ginsenosides can be divided into protopanaxadiol (PPD) and protopanaxatriol (PPT) according to their chemical structure [8,9,10]. Among them, PPD-type saponins include Ra1, Ra2, Ra3, Rb1, Rb2, Rb3, Rc, Rd, Rg3, F2, Rh2, Rs1, Rs2, Rs3, and other monomeric saponins; PPT-type saponins include Re, Rf, Rg1, Rg2, Rh1, F1, F3, F5, and other monomeric saponins [9]. The functions of ginsenoside are diverse [6], but the progress of ginsenoside accumulation is influenced by many factors, hence, the relevant content and type of different tissues encompass many varieties. Because of the long growing cycle of ginseng, the application of ginsenoside is limited in the field of treatment, and application must be in accordance with the long growing cycle of ginseng. It has been reported that the pathway of ginseng synthesis is very complex because the genes are diverse in spatiotemporal expression patterns. Lots of researchers perform deep research, but their studies have focused on the research and verification of certain genes or single genes [11,12,13]. Although some results have been achieved, it is still far from enough to explain the overall substance metabolism pathway. Still, researchers rarely focus on the entire Rb3 genes; therefore, we have mainly concentrated on complete ginsenoside Rb3 gene analysis and study.

Ginsenoside Rb3 belongs to the ginseng diol-type saponin, the molecular formula is C_53_H_90_O_22_, and the molecular weight is 1079.27 [14]. Studies have shown that ginsenoside Rb3 has important physiological effects on the immune system, nervous system, inflammatory responses, cardiovascular system, blood system, and other aspects [15,16,17,18,19] of human health. With additional research, its various biological effects and pharmacological mechanism of action will be gradually revealed, and the application of ginsenoside Rb3 may become a new treatment for the nervous system, cardiovascular system, blood system, and viral diseases [5,20]. Although ginsenoside Rb3 has important effects, its synthesis and metabolism in plants are not thoroughly studied, so its analysis and verification on the basis of omics will help explain its synthesis and mechanism of action and also provide reference for the study of other monomer saponins.

Methyl jasmonate (MeJA) is a phytohormone that is ubiquitous in plants; it regulates various physiological activities in plants, such as defense responses, flowering, and senescence [21]. MeJA is the main way for plants to resist stress. In recent years, studies have found that MeJA appears after the plant is attacked by pathogens, causing the formation of reactive oxygen species (ROS). Many reports have proven that the addition of MeJA can increase the content of ginsenosides in ginseng hairy roots [22,23].

Here, we chose some genes that have a great contribution to the synthesis of ginsenoside Rb3. Through a MeJA treatment experiment, we found that *PgRb3S01*, *PgRb3S03*, and *PgRb3S10* genes were downregulated at the beginning of the treatment but significantly increased with increases in induction time. This study is a reference for revealing the expression rules of ginsenoside Rb3 synthesis-related genes and elucidating the regulatory mechanism of MeJA and lays a theoretical foundation for research into ginsenoside Rb3 synthesis.

## 2. Materials and Methods

### 2.1. Basic Analysis of Ginsenoside Rb3 Synthesis-Related Genes

An NCBI Conserved Domains Search was used to conduct a conservative domain search (https://www.ncbi.nlm.nih.gov/Structure/cd/wrpsb.cgi, accessed on 1 October 2016) to initially determine the function of ginsenoside Rb3 synthesis-related candidate genes, and the NCBI ORF Finder (https://www.ncbi.nlm.nih.gov/orffinder/, accessed on 13 October 2016) was used to find its open reading frame to determine whether the sequence was to be expressed as a functional protein. Then, through Blast (https://blast.ncbi.nlm.nih.gov/Blast.cgi, accessed on 3 November 2016), we checked if there were similar proteins that had been reported and identified.

### 2.2. Screening of Candidate Genes Related to Ginsenoside Rb3 Synthesis

The expression levels of ginsenoside Rb3 synthesis-related gene groups (in the passage called *PgRb3* genes) were divided into two groups from low to high and input into SPSS Version 23.0 software together with the corresponding ginsenoside Rb3 content data for independent sample t-test analysis. According to the results, the genes whose gene expression level changed significantly relative to the content of saponins were selected as candidate genes related to ginsenoside Rb3 synthesis for further analysis.

### 2.3. GO (Gene Ontology) Annotation for Candidate Genes Related to Ginsenoside Rb3 Synthesis

Blast, mapping, annotation, and KEGG analysis of the candidate genes related to ginsenoside Rb3 synthesis were performed in sequence using Blast2GO Version 5.0 software [24] to obtain the annotation information and metabolic pathway information.

### 2.4. Enrichment Analysis of Candidate Genes Related to Ginsenoside Rb3 Synthesis

Based on the GO annotation results of the candidate genes related to ginsenoside Rb3 synthesis (*PgRb3*), the chi-square test method and enrichment significance analysis were performed on each gene under each function to obtain their enrichment rules.

### 2.5. Analysis of Spatiotemporal Expression Patterns of PgRb3 Genes

The R programming language (http://www.rproject.org/, accessed on 21 November 2016) and TBtools software version 1.082 [25] was used to visualize the expression of *PgRb3* genes as heatmaps to obtain their expression characteristics in 14 different tissues of 4-year-old ginseng roots, from 4 different growing-years (5, 12, 18, and 25 years), and 42 farmers’ cultivars of 4-year-old ginseng roots. In addition, we used the R language to calculate the Pearson correlation coefficient between each *PgRb3* gene pair. Then we used the BioLayout Express ^3D^ Version 3.3 software [26] to visualize the results as an interactive network graph.

### 2.6. Correlation Analysis of PgRb3 Genes and Saponin Content

SPSS Version 23.0 software was used to calculate the correlation between the expression levels of ginsenoside Rb3 synthesis-related candidate *PgRb3* genes and the content of different monomer saponins. In addition, we sorted the ginsenoside Rb3 content and divided them into three groups: high, medium, and low, and selected Rb3 synthesis-related candidate genes that were significantly related to the changes in the saponin content of different components.

### 2.7. Correlation Analysis of PgRb3 Genes and Known Key Enzyme Genes for Ginsenoside Synthesis

Based on the above correlation analysis 2.6 results, combined with the previous gene function annotation results, we screened out the known key enzyme genes that were most closely related to ginsenoside Rb3 synthesis and predicted to have functions. We had previously cloned 20 known ginsenoside-biosynthesis key enzyme genes, including *SS* (*SS_1*) [27], SE (*SE2_1*, *SE2_2*, and *SE2_4*) [28], DS (*DS_1*, and *DS_3*) [29], β-AS (*AS_3*, *AS_6*, *AS_7*, and *AS_14*) [30], FPS (*FPS_22*) [31], CAS (*CAS_11*, *CAS_13*, *CAS_14*, *CAS_17*, and *CAS_23*) [32], UGT (*UGT71A27_2*) [33], and three CYP (*CYP716A47_1*, *CYP716A53v2_1*, and *CYP716A52v2_3*) genes [34,35,36].

### 2.8. Expressions of PgRb3 Genes in Ginseng Hairy Roots by MeJA Treatment

Inoculate 0.2 g of ginseng hairy roots in a 250 mL Erlenmeyer flask containing 150 mL of 1/2 MS liquid medium and place in a shaker for cultivation at 22 °C with shaking at 110 rpm. During the mid-logarithmic phase (Day 23), add methyl jasmonate (MeJA) to induce. Use three replicates and one control for each experimental group. The amount of MeJA added is 200 μM, and the duration is 6, 12, 24, 48, 72, 96, and 120 h. At each time-point, harvest three biologically duplicated samples and one blank control with no MeJA treatment. Flash-freeze the ginseng hairy root samples in liquid nitrogen for RNA extraction [37].

Use qRT-PCR with the cDNA of ginseng hairy roots, using MeJA treatment and the *GAPDH* gene (GenBank Accession No. KF699323) from *Panax ginseng* as the reference using SYBR Premix Ex Taq II (Tli RNaseH Plus) (TaKaRa, Dalian, China). The reaction system should include 5.0 μL SYBR Premix Ex Taq II, 0.2 μL Rox Ⅱ, 0.4 μL designed primers (Table 1), 0.5 μL cDNA and 3.5 μL RNase-free water. Perform the reaction as follows: predenaturation at 95 °C for 30 s; PCR for 40 cycles at 95 °C for 5 s and 60 °C for 34 s; melting curve at 95 °C for 15 s, 60 °C for 60 s, and 95 °C for 15 s. Analyze the results using the 2^−ΔΔCt^ method [38,39].

## 3. Results

### 3.1. Basic Analysis of Candidate Gene Population Related to Ginsenoside Rb3 Synthesis

To obtain a profile of interested ginsenoside Rb3 synthesis-related genes for its spatiotemporal expression, we conducted preliminary bioinformatics analyses on the transcriptome of *Panax ginseng*. Based on the existing transcriptome data of Jilin ginseng [11,40], after using the gene expression and trait association analysis methods, candidate genes related to the synthesis of ginsenoside Rb3 were obtained. Their lengths vary from 211 bp to 2603 bp, and their average length is 949 bp. Except for one that does not have a complete open reading frame, all others contain complete open reading frames, encoded amino acids of which range from 35 to 690. Among them, 14 genes have conserved domains, 13 genes relate to proteins with protein-encoding functions, and the conserved domains of 7 genes relate to enzymes. This basic information is shown in Table S1 (in the supplementary materials).

### 3.2. Screening of Candidate Genes Related to Ginsenoside Rb3 Synthesis

To preliminarily obtain the candidate genes involved in ginsenoside Rb3 synthesis, we grouped the genes according to their expression levels and entered the corresponding ginsenoside Rb3 content into SPSS software for independent sample t-test analysis, then we screened out 18 candidate genes related to ginsenoside Rb3 synthesis for subsequent systematic analysis. The 18 genes screened out above were renamed in order, and the naming method was *PgRb3S* + sequence number in Table 2. The specific analysis results are shown in Table S2 (in the supplementary materials).

### 3.3. Annotation of GO Function for Candidate Genes Related to Ginsenoside Rb3 Synthesis

**Figure 1:**
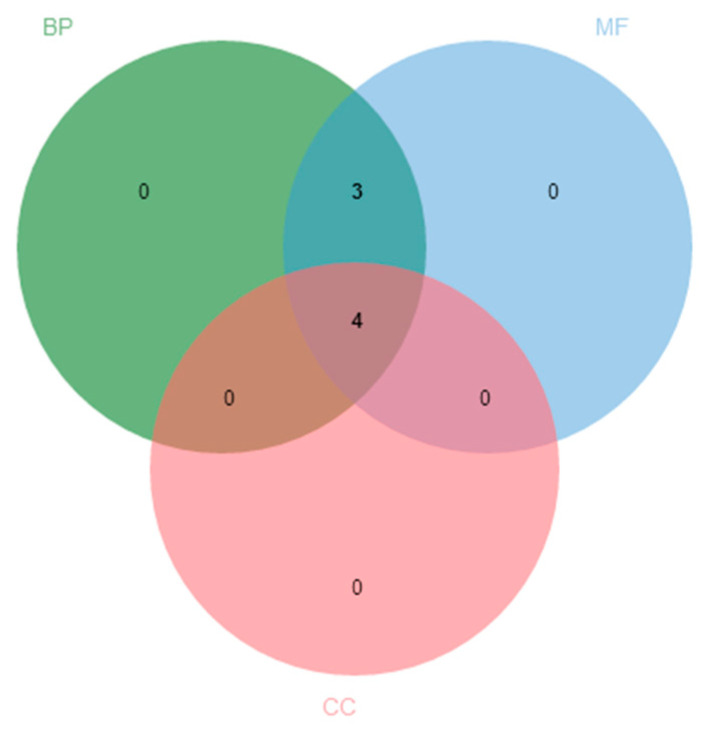
GO annotation functional classification of ginsenoside Rb3 synthesis-related candidate genes. The Venn chart of the numbers of 18 candidate genes related to ginsenoside Rb3 synthesis.

**Figure 2:**
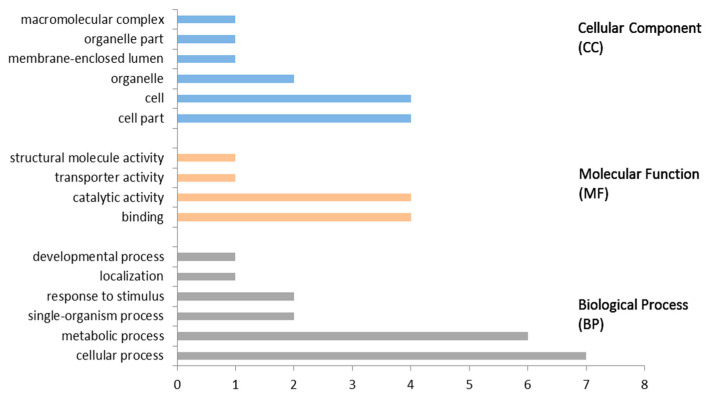
GO annotation functional classification of ginsenoside Rb3 synthesis-related candidate genes on level 2. The ginsenoside Rb3 synthesis-related candidate genes are classified into 16 functional categories at level 2, including six CC-functional categories (blue), four MF-functional category (orange), and six BP-functional categories (gray).

To provide the function information for each candidate genes for further study, we used the Blast2GO software to perform GO function annotation on 18 candidate genes related to ginsenoside Rb3 synthesis. The results are shown in Figure 1. Only seven sequences were annotated to functions, four of which were involved in all three functions, and three of which were involved in molecular functions (MF) and biological processes (BP). No genes were involved in only a single functional field, indicating that the functional direction of this gene group is diverse. Although the number of annotated sequences is small and does not exceed half of the total number, the specific functions annotated are very wide, as shown in Figure 2, including catalytic activity (GO:0003824), binding (GO:0005488), transporter activity (GO:0005215), structural molecule activity (GO:0005198), cellular process (GO:0009987), metabolic process (GO:0008152), response to stimulus (GO:0050896), single-organism process (GO:0044699), localization (GO:0051179), developmental process (GO:0032502), macromolecular complex (GO:0005575), organelle part (GO:0044422), cell (GO:0005623), organelle (GO:0043226), cell part (GO:0044464), and membrane-enclosed lumen (GO:0031974). The above results indicate that the functions of single genes related to ginsenoside Rb3 synthesis are diverse.

### 3.4. Enrichment Analysis of GO Annotation for PgRb3 Genes

**Figure 3:**
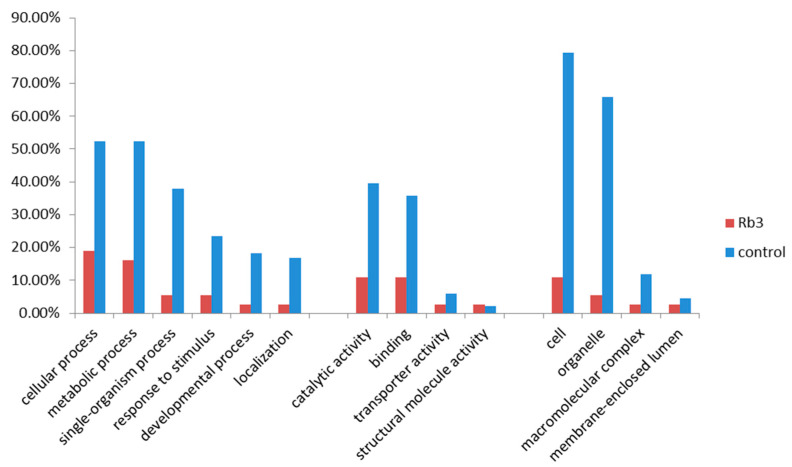
Enrichment analysis of GO annotation functions of *PgRb3* genes on level 2 using the chi-square test. The GO term categorization of the Jilin ginseng total transcriptome in 14 tissues of the four-year-old ginseng used for the identification of the *PgRb3* genes as the background control for the enrichment analysis.

To further identify the functions on which *PgRb3* genes mainly focus, we performed a chi-square test on the annotation results of *PgRb3* genes and the annotation results of Jilin ginseng total transcriptome in 14 tissues of the four-year-old ginseng from our laboratory [11]. The results are shown in Figure 3. In all the functions annotated by ginsenoside Rb3 synthesis-related candidate genes, the annotation ratio is lower than the annotation ratio of the total transcriptome, but in transporter activity (GO: 0005215), structural molecule activity (GO: 000519), and membrane-enclosed lumen (GO: 0031974), the annotation ratio of the three functions is similar to the total transcriptome, indicating that ginsenoside Rb3 synthesis-related genes have relatively concentrated functions in these three functions.

### 3.5. Heatmap Analysis for Spatiotemporal Expression Patterns of PgRb3 Genes

**Figure 4:**
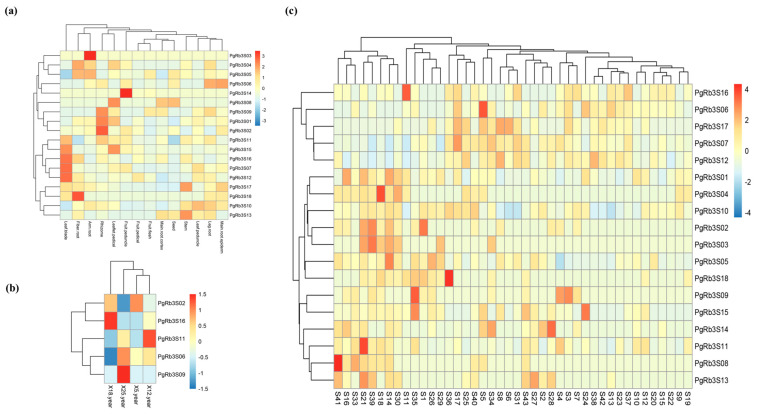
Heatmap analysis spatiotemporal expression patterns of *PgRb3* genes in *Panax ginseng*. (**a**) The *PgRb3* genes expressed in the 14 different tissues of four-year-old ginseng. (**b**) The *PgRb3* genes expressed in the four different growing-years (5, 12, 18, and 25 years) of ginseng roots. (**c**) The *PgRb3* genes expressed in the 42 farmers’ cultivars of four-year-old ginseng roots.

To make it easier to understand the interested genes’ spatiotemporal expression characteristics, we used TBtools software to visualize the expression levels of *PgRb3* genes expressed in 14 tissues, in four different growing-years of ginseng roots, and 42 farmers’ cultivars of ginseng roots as heatmaps. The results are shown in Table S3 (in the supplementary materials), Figure 4. All 18 candidate genes related to ginsenoside Rb3 synthesis were expressed in 14 different tissues, and the same gene expressed differently in different tissues. Among them, *PgRb3S03*, *PgRb3S14*, and *PgRb3S18* genes were highly expressed in arm root, fruit peduncle, and fiber root, respectively. The expression of the same gene was also different in farmer’s cultivars, of which *PgRb3S04*, *PgRb3S06*, *PgRb3S08*, *PgRb3S09*, *PgRb3S10*, *PgRb3S11*, and *PgRb3S16* genes were high expressed in only a certain genotype. In the four different growing-years of ginseng root, only five *PgRb3* genes were expressed, and the expression was similar. The above results indicate that the *PgRb3* gene has spatiotemporal specificity.

### 3.6. Network Analysis for Spatiotemporal Expression Patterns of PgRb3 Genes

**Figure 5:**
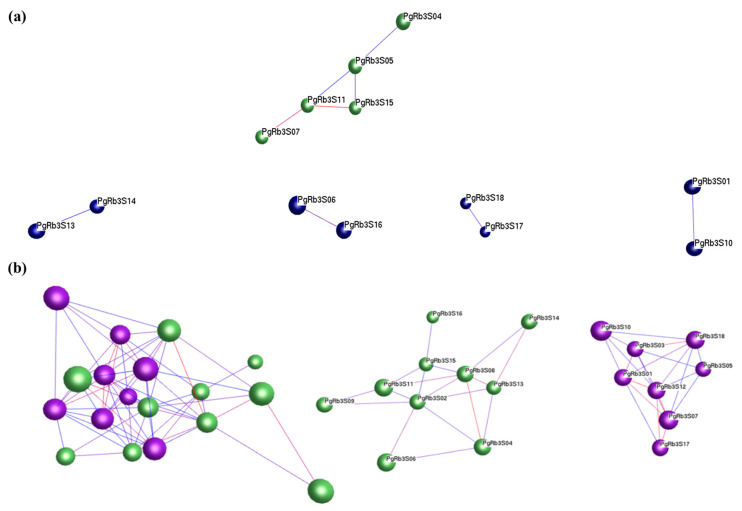
Network analysis of spatiotemporal expression patterns of *PgRb3* genes in *Panax ginseng,* constructed at *P ≤ 0.05*. (**a**) The *PgRb3* genes analyze the network in the 14 different tissues of four-year-old ginseng. (**b**) The *PgRb3* genes analyze the network in the 42 farmers’ cultivars of four-year-old ginseng roots. The nodes are *PgRb3* genes, and the edges are interaction. The *PgRb3* genes are in the network of clusters.

To elucidate the interaction among interested genes, we conducted the network analysis from interested-gene-to-interested-gene perspective. For the expression levels of ginsenoside Rb3 synthesis-related candidate genes expressed in 14 tissues, four different growing-years of ginseng roots, and 42 farmers’ cultivars of ginseng roots, we calculated the Pearson correlation coefficient using the R language and then visualized it with BioLayout express 3D software as an interaction network diagram in Figure 5. In 14 tissues, the *PgRb3* genes related to ginsenoside Rb3 synthesis expressed in different tissues did not form an entire interaction network, and only five of them constituted a small interaction network (*P ≤ 0.05*). In 42 farmers’ cultivars of ginseng roots, when *P ≤ 0.05*, the *PgRb3* genes constitute an interaction network with 2 clusters, 10 nodes, and 19 edges, indicating that *PgRb3* genes related to ginsenosides Rb3 synthesis expressed in different cultivars have significant interactions. In four different growing-years of ginseng roots, only five *PgRb3* genes were expressed, and no interaction network was formed at a significant level. The *PgRb3* genes related to the synthesis of ginsenoside Rb3 involved in this study were selected based on the content of ginsenoside Rb3, and the content of saponin was determined in the ginseng roots of 42 farmers’ cultivars. Hence, a significant interaction network was formed from the data of 42 farmers’ cultivars. However, no significant interaction network was formed from data of 14 tissues or four different growing-years of ginseng roots.

### 3.7. Correlation Analysis of PgRb3 Genes Expression and Ginsenoside Content

**Figure 6:**
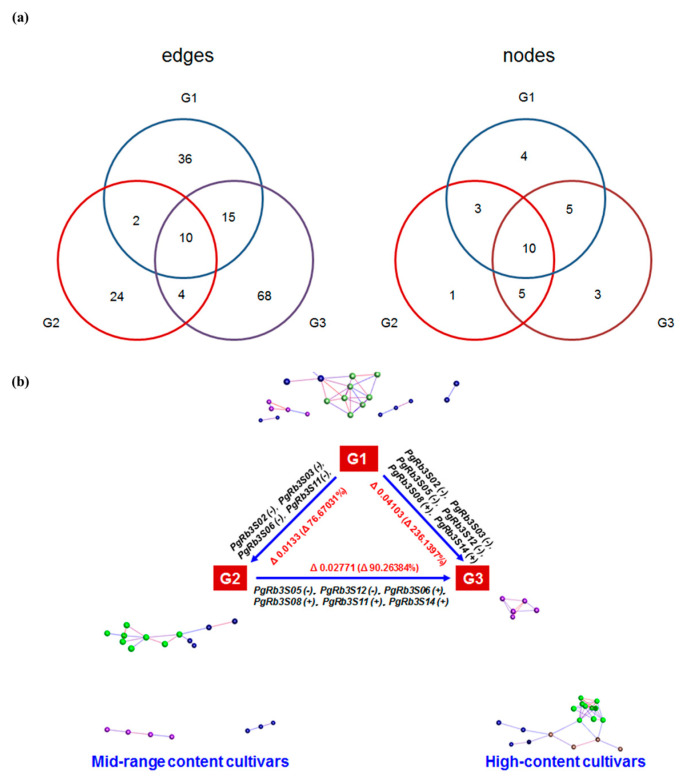
Correlation analysis of *PgRb3* gene expression and ginsenoside content in *Panax ginseng*. (**a**) The *PgRb3* genes’ frequency as nodes and number of their edges in each network. (**b**) Correlation analysis of *PgRb3* genes and Rb3 content level changes.

To elucidate the interaction among interested genes and saponins, we conducted a network analysis from the gene-to-saponin content perspective. A Pearson correlation analysis was performed on the expression levels of *PgRb3* genes related to ginsenoside Rb3 synthesis and the content of different monomer saponins. The results are shown in. Among all 18 genes, except for *PgRb3S07* and *PgRb3S11*, the other 16 genes were significantly related to the content of different monomer saponins, of which *PgRb3S01* gene was significantly related to the content of six monomer saponins, indicating that it may be important in the process of ginsenoside synthesis. Additionally, according to the content of ginsenoside Rb3, we divided the different cultivars into three groups: low content (G1), medium content (G2), and high content (G3), and built an interaction network in each group, and for each gene, we counted its frequency as nodes and number of its edges in each network in Figure 6a. Among the 18 genes, 10 genes are nodes in three groups. Overall, these genes have the largest number of edges in the G3 group, indicating that the close relationship between the above genes is positively correlated with saponin content. We counted the node genes in the above network and looked at the ones that changed with the content in Figure 6b, where △ represents the difference in saponin content between different groups, and △% represents the growth rate of saponin content from the low-content group to the high-content group. From the low saponin-content group to the high saponin-content group, the genes that changed corresponding were *PgRb3S02*, *PgRb3S03*, *PgRb3S05*, *PgRb3S06*, *PgRb3S08*, *PgRb3S11*, *PgRb3S12*, and *PgRb3S14*, indicating that the above genes played important roles in regulating the content of ginsenoside Rb3 effect.

### 3.8. Correlation Analysis of PgRb3 Genes and Known Ginsenoside Synthesis Key Enzyme Genes

**Figure 7:**
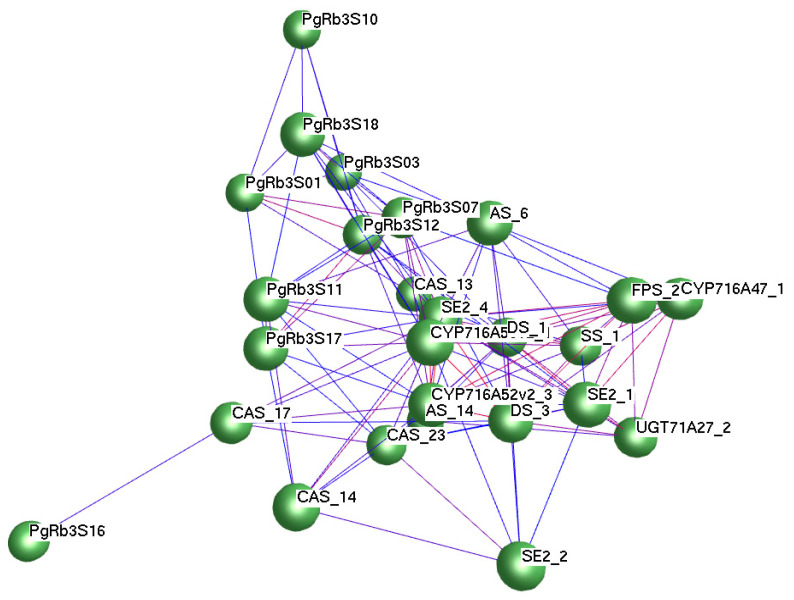
Network analysis of *PgRb3* genes and ginsenoside-biosynthesis key enzyme genes in *Panax ginseng*. Green nodes were genes that include 9 *PgRb3* genes and 17 ginsenoside-biosynthesis key enzyme genes. *PgRb3* genes include *PgRb3S01*, *PgRb3S03*, *PgRb3S07*, *PgRb3S10*, *PgRb3S11*, *PgRb3S12*, *PgRb3S16*, *PgRb3S17*, and *PgRb3S17*; ginsenoside-biosynthesis key enzyme genes include *FPS_22*, *DS_1*, *DS_3*, *SE2_4*, *SE2_1*, *SE2_2*, *SS_1*, *AS_6*, *AS_14*, *CAS_13*, *CAS_14*, *CAS_17*, *CAS_23*, *CYP716A53v2_1*, *CYP716A52v2_3*, *CYP716A47_1*, and *UGT71A27_2.*

To elucidate the interaction among interested genes and known ginsenoside-synthesis key enzyme genes, we conducted a network analysis from an interested-gene-to-known-key-enzyme-gene perspective. We established a network of *PgRb3* genes and known key enzyme genes for ginsenoside synthesis. There are 17 known key enzyme genes (*FPS_22*, *DS_1*, *DS_3*, *SE2_4*, *SE2_1*, *SE2_2*, *SS_1*, *AS_6*, *AS_14*, *CAS_13*, *CAS_14*, *CAS_17*, *CAS_23*, *CYP716A53v2_1*, *CYP716A52v2_3*, *CYP716A47_1*, *UGT71A27_2*) [40,41] forming an interaction network, and the network also contains nine *PgRb3* genes related to ginsenoside Rb3 synthesis, including *PgRb3S01*, *PgRb3S03*, *PgRb3S07*, *PgRb3S10*, *PgRb3S11*, *PgRb3S12*, *PgRb3S16*, *PgRb3S17*, and *PgRb3S17*. Correlation analysis was performed on the expression levels of all key enzyme genes in the network and nine *PgRb3* genes related to ginsenoside Rb3 synthesis (Figure 7). The results are shown in Table S4 (in the supplementary materials). A total of seven genes including *PgRb3S01*, *PgRb3S03*, *PgRb3S07*, *PgRb3S10*, *PgRb3S11*, *PgRb3S17*, and *PgRb3S18* genes were significantly related to different key enzyme genes, indicating that these genes play an important role in the synthesis of ginsenoside Rb3.

### 3.9. Identification of PgRb3 Genes Related to Ginsenoside Rb3 Synthesis in Panax ginseng

**Figure 8:**
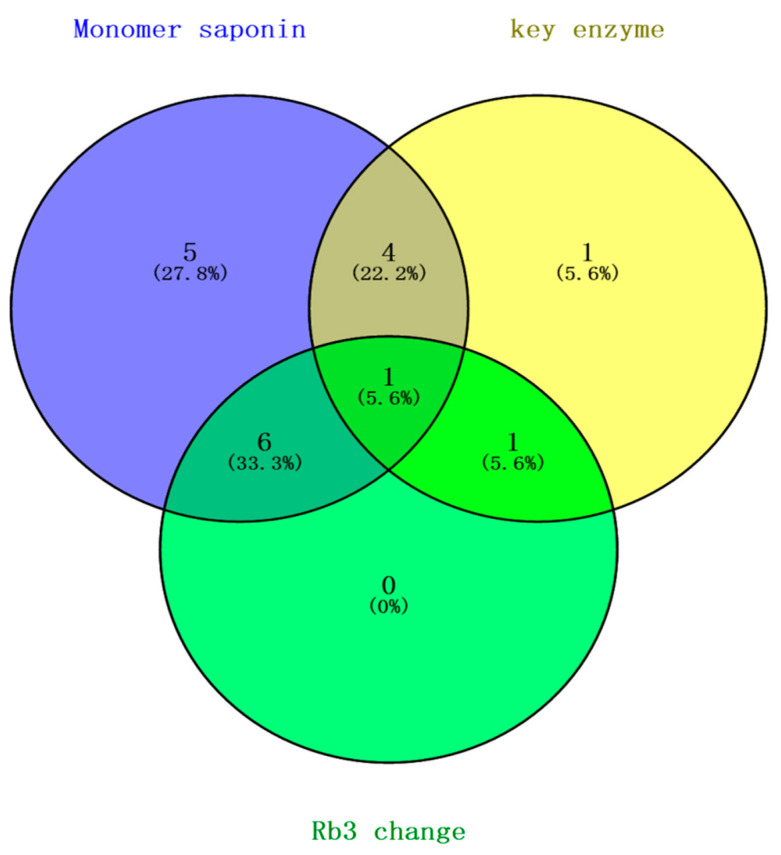
Identification of *PgRb3* genes related to ginsenoside Rb3 synthesis in *Panax ginseng*. Blue is the result of a t-test analysis of *PgRb3* gene expression and Rb3 saponin content; yellow is the result of a network analysis of *PgRb3* genes and ginsenoside biosynthesis key enzyme genes; green is the result of a correlation analysis of *PgRb3* genes and Rb3 content level changes.

Finally, to integrate the analysis results from all of the above three perspectives, we performed statistics on those three results (Figure 8). One gene (*PgRb3S03*) was significantly correlated in all analyses, and another 11 genes were significantly correlated in two analyses, indicating that the above 12 genes are highly correlated with the synthesis of ginsenoside Rb3. Combining this result with previous gene function analysis, we finally selected seven genes that are predicted to have important functions in the synthesis of ginsenoside Rb3. Combining this with the preliminary analysis results, we finally selected *PgRb3S01*, *PgRb3S03*, and *PgRb3S10* for qRT-PCR verification. Among them, the annotation function of the *PgRb3S03* gene is the DNA mismatch repair ATPase MutL. Although it is not related to the secondary metabolic process, it is significantly related to ginsenosides in multiple levels of correlation, so it was selected for verification; the *PgRb3S01* gene is the mitochondrial dicarboxylic acid transport protein, which may be related to plant cell material transport; the *PgRb3S10* gene is an RNA binding protein, which may be related to plant stress resistance and hormone response.

### 3.10. Study on PgRb3 Gene Expression Regularity under MeJA Treatment

**Figure 9:**
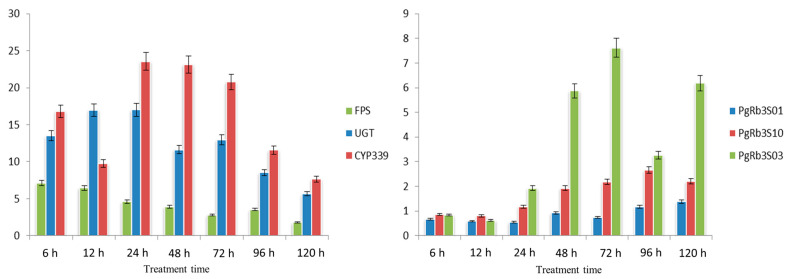
Expression changes of related genes after MeJA treatment. (**a**) Relative expression levels of the three ginsenoside-biosynthesis key enzyme genes correlated with *GADPH* from ginseng as the reference gene. (**b**) Relative expression levels of the three *PgRb3* genes correlated with *GADPH* from ginseng as the reference gene.

As shown in Figure 9, under the treatment of MeJA, the expression levels of key enzyme genes *FPS* (*FPS_22*), *UGT* (*UGT71A27_2*), and *CYP339* (*CYP716A47_1*) were significantly upregulated relative to the control group and showed a trend of increasing first and then, decreasing after MeJA treatment. Among them, the expression level of the *FPS* gene reached its peak at 6 h of induction, and its expression level was 7.1 times that of the control group; the expression level of the *CYP339* gene reached its peak at 24 h, and its expression level was 25 times that of the control group; UGT’s peak was at 24 h, which was 17 times the expression of the control group. Among the genes related to ginsenoside Rb3 synthesis, *PgRb3S01*, *PgRb3S03*, and *PgRb3S10* genes were downregulated at the beginning of the treatment, but this significantly increased with the increase in induction time. The expression of the *PgRb3S03* gene reached 7.6 times that of the control group at 72 h, and the expression of the *PgRb3S10* gene reached 2.6 times that of the control group at 96 h, and the expression of the *PgRb3S01* gene reached 1.3 times that of the control group at 120 h. This result not only confirmed the inducing effect of methyl jasmonate on the content of saponins in ginseng hair roots from the perspective of gene expression but also confirmed the reliability of the results obtained by the systematic analysis of this study. The genes we selected through analysis did participate in the synthesis of ginsenosides, and the expression of these genes was time-specific, in the order *FPS* → *UGT*, *CYP339* → *PgRb3S03* → *PgRb3S10* → *PgRb3S01*. This result provides a theoretical basis for the study of the ginsenoside Rb3 synthesis pathway.

## 4. Discussion

Ginsenosides are secondary metabolites of plants. Their synthesis and metabolism are regulated by the entire plant. In the process of saponin formation, a series of structural genes and regulatory genes are involved. Previous studies have focused on the research and verification of certain genes or single genes. Although some results have been achieved, it is still far from enough to explain the overall substance metabolism pathway. Therefore, it is very important to conduct an integrated and systematic analysis and verification of all genes involved in the synthesis of a substance.

The *PgRb3* genes involved in the synthesis of ginsenoside Rb3 in this study include many genes encoding enzymes, some regulatory genes, and some gene fragments with unknown functions. It is nonviable to directly explain the role and relationship of all of these genes without any relevant research. When the expression of a gene changes and the content of monomer saponins change significantly, we can speculate that the gene contributes to the change of the content of monomer saponins, but we cannot rule out that the gene controls other related traits, instead of the possibility of monomer saponin synthesis, so we also introduced the key enzyme gene of the verified saponin synthesis as evidence to study its correlation with the target gene. Since ginsenosides are composed of aglycones, and different monomer saponins can convert to each other, we conducted a correlation analysis among the ginsenoside Rb3 synthesis-related genes and other saponin content changes to corroborate the gene function. In summary, when a gene satisfies all the necessary conditions, its expression level being significantly related to the content change of ginsenoside Rb3, its expression level being significantly related to the content change of other monomer saponins, and its expression level being significantly correlated with the expression level of known key enzymes for saponin synthesis, then we can determine that this gene is related to ginsenoside Rb3 synthesis.

We selected *FPS*, *UGT*(*UGT71A27_2*), and *CYP339* (*CYP716A 47_1*) as the representatives of key enzyme genes for ginsenoside synthesis and *PgRb3S01*, *PgRb3S03*, and *PgRb3S10* as the representatives of ginsenoside Rb3 synthesis-related genes to study their expression patterns under the treatment of elicitors, which provides the basis for future study on ginsenoside Rb3 synthesis and transport pathways. *FPS* is the key enzyme gene before aglycon synthesis. *UGT* and *CYP* are the key enzyme genes for monomeric saponin synthesis. *PgRb3S01* codes for the mitochondrial dicarboxylic acid transporter, and PgRb3S03 is responsible for the mismatch repairment. *PgRb3S10* codes for the RNA binding protein. The results of this study showed that the expression sequence of these genes under MeJA elicitation is *FPS*→ *UGT*, *CYP339*→ *PgRb3S03*→ *PgRb3S10*→ *PgRb3S01*. We speculated that this sequence represents the material metabolism process of Rb3 synthesis and transport. To rephrase it, the events involved in Rb3 synthesis and transport occur in order as follows: aglycon synthesis → monomer saponin synthesis → acting on DNA → acting on RNA → obtaining energy for transfer. A future study may focus on these specific mechanisms, regulatory functions, and interrelationships.

In this study, the gene groups related to the traits were screened from the transcriptome for systematic analysis, which has the advantages of wide coverage, comprehensive information, and rapid analysis. It provides more information for the study of genes related to ginsenoside synthesis, and it has great advantages in explaining the ginsenoside synthesis pathway, metabolic network, and interaction among genes.

## 5. Conclusions

In summary, we initially screened out 18 *PgRb3* genes related to ginseng Rb3 synthesis. Then we conducted a preliminary analysis of these 18 genes and found that they have functional diversity and expression spatiotemporal specificity. Through correlation analysis of multiple perspectives, we finally selected seven genes that have a great contribution to the synthesis of ginsenoside Rb3. Through a MeJA treatment experiment, we found that *PgRb3S01*, *PgRb3S03*, and *PgRb3S10* genes were downregulated at the beginning of the treatment but significantly increased with increases in induction time, which proved that the previous systematic analysis results were credible. This project lays a theoretical foundation for the synthesis and metabolism mechanism of ginsenoside Rb3. It also provides a reference for the synthesis mechanism of the ginsenoside monomer and a theoretical basis for the factory production of ginsenoside Rb3.

## Figures and Tables

**Table 1:** Sequences of the qRT-PCR primers for *PgRb3* genes, *UGT*, *CYP450*, *FPS,* and *GADPH* gene primers.

Gene Name	Forward Primer (5’-3’)	Reverse Primer (5’-3’)
*PgRb3S01*	CAATGAAAACCCTGAAACTCG	TAGGAGTATGAGGCAGAGGTAAAG
*PgRb3S03*	TGGTCGGAGATCCACTTGCT	TTATTCATGCTGCTCATCTGATCTT
*PgRb3S10*	GAAGATGGTCAAGGGACTGCT	TGTCGTCACCCACTTGCCT
*UGT* *(UGT71A27_2)*	TGCGTCCGTCTATCCCTAAAG	TGATGTCCTGTCCAAGAATCCTAC
*CYP339* *(CYP716A47_1)*	GCAGAGGTTTACTTTGGCAC	TCACATTGATGGAGAGGACAC
*FPS*	GGATGATTATCTGGATTGCTTTGGCGAG	CAGTGCTTTTACTACCAACCAGGAG
*GADPH*	TTCCCACTGTGGATGTC	CTCCGACTCCTCCTTGATAGC

**Table 2:** The 18 ginsenoside Rb3 synthesis-related candidate genes that were renamed in this study.

Number	Gene ID	Named ID
1	*comp122476_c0_seq1*	*PgRb3S01*
2	*comp34816_c0_seq1*	*PgRb3S02*
3	*comp43937_c0_seq2*	*PgRb3S03*
4	*comp439496_c0_seq1*	*PgRb3S04*
5	*comp47140_c0_seq1*	*PgRb3S05*
6	*comp48914_c0_seq3*	*PgRb3S06*
7	*comp50009_c0_seq2*	*PgRb3S07*
8	*comp52220_c0_seq2*	*PgRb3S08*
9	*comp61172_c1_seq1*	*PgRb3S09*
10	*comp61669_c1_seq3*	*PgRb3S10*
11	*comp63877_c0_seq2*	*PgRb3S11*
12	*comp64020_c0_seq3*	*PgRb3S12*
13	*comp64616_c1_seq6*	*PgRb3S13*
14	*comp65415_c0_seq45*	*PgRb3S14*
15	*comp65868_c1_seq1*	*PgRb3S15*
16	*comp65985_c1_seq26*	*PgRb3S16*
17	*comp66742_c1_seq1*	*PgRb3S17*
18	*comp709833_c0_seq1*	*PgRb3S18*

## Data Availability

The sequences of the *PgRb3* genes will be submitted to NCBI under BioProject RJNA302556.

## Supplementary Materials

The following are available online at https://www.mdpi.com/article/10.3390/life11050387/s1, Table S1: basic information on ginsenoside Rb3 synthesis-related candidate genes. Table S2: t-test analysis of ginsenoside Rb3 synthesis-related candidate gene expression and Rb3 saponin content. Table S3: the expressions of PgRb3 genes (TPM) in 42 farmers’ cultivars, 14 tissues, and 4 different years-old ginseng. Table S4: correlation analysis of the expression of PgRb3 genes and known ginsenoside-synthesis key enzyme genes in *Panax ginseng*.
